# Modeling Nanoparticle Targeting to a Vascular Surface in Shear Flow Through Diffusive Particle Dynamics

**DOI:** 10.1186/s11671-015-0942-z

**Published:** 2015-05-27

**Authors:** Bei Peng, Yang Liu, Yihua Zhou, Longxiang Yang, Guocheng Zhang, Yaling Liu

**Affiliations:** School of Mechatronics Engineering, University of Electronic Science and Technology of China, Chengdu, 611731 China; Center for Robotics, University of Electronic Science and Technology of China, Chengdu, 611731 China; Department of Mechanical Engineering and Mechanics, Lehigh University, Bethlehem, PA 18015 USA; Bioengineering Group, Lehigh University, Bethlehem, PA 18015 USA

**Keywords:** Coarse-grained molecular dynamics, Capillary, Shear flow, Nanoparticle binding

## Abstract

Nanoparticles are regarded as promising carriers for targeted drug delivery and imaging probes. A fundamental understanding of the dynamics of polymeric nanoparticle targeting to receptor-coated vascular surfaces is therefore of great importance to enhance the design of nanoparticles toward improving binding ability. Although the effects of particle size and shear flow on the binding of nanoparticles to a vessel wall have been studied at the particulate level, a computational model to investigate the details of the binding process at the molecular level has not been developed. In this research, dissipative particle dynamics simulations are used to study nanoparticles with diameters of several nanometers binding to receptors on vascular surfaces under shear flow. Interestingly, shear flow velocities ranging from 0 to 2000 s^−1^ had no effect on the attachment process of nanoparticles very close to the capillary wall. Increased binding energy between the ligands and wall caused a corresponding linear increase in bonding ability. Our simulations also indicated that larger nanoparticles and those of rod shape with a higher aspect ratio have better binding ability than those of smaller size or rounder shape.

## Background

Nanoparticulate systems have been widely used for drug and gene delivery, imaging, and photodynamic therapy [1–12]. A typical nanoparticulate system consists of a nanoplatform, such as liposomes, polymeric micelles, quantum dots, nanoshells, or dendrimers, coated with ligands like hydrophobic drugs, DNA, or imaging agent. Ligands direct the nanoplatforms to specific locations and help to improve their bioavailability during circulation in a biological system [2, 3, 7–10, 13, 14]. Two main methods are used to transport ligand-coated nanoparticles (NPs) to diseased sites: passive and active targeting. In passive targeting, the accumulation of NPs is achieved by the enhanced permeability and retention effect [3, 7, 10, 15, 16] because the leaky vasculature and low lymphatic drainage prolong the residence time of NPs in the tumor. Conversely, active targeting is mediated by specific interactions between ligands that are connected via flexible spring tethers and receptors that are overexpressed at the pathological site. The highly concentrated receptors around pathological sites are preferred for ligand interaction because they can enhance NP internalization and retention [3, 7, 10, 15, 16].

Understanding the effects of NP size, hydrodynamic force, and multivalent interactions with a targeted biosurface on the mechanisms of a targeted delivery process is essential to aid the design and fabrication of NP systems [15]. Experimental techniques, such as fluorescence spectroscopy combined with microfluidics [17] and surface plasmon resonance [18], have been developed to investigate the ligand–receptor binding kinetics in vivo. The acquired experimental data indicate that the process of NP binding to a targeted surface is a synergic result of many factors, including the shape and diffusion of NPs, the flow effects [17, 19], as well as binding and internalization kinetics [20]. However, exploring this phenomenon experimentally is a very time-consuming task because of the small size of NPs and the dynamic nature of the transportation–deposition process; moreover, many details are difficult to capture because the binding process is very fast.

Therefore, theoretical modeling and numerical simulation have been performed to study the margination and adhesion processes of NPs in a fluid. For instance, Liu et al. [21] investigated the shape-dependent adhesion kinetics of non-spherical NPs through theoretical modeling. The influences of NP shape, ligand density, and shear rate on bonding ability under Brownian dynamics were systematically studied. They also investigated the distribution of NPs with different shapes and sizes in a mimetic branched blood vessel and found that NPs with smaller size and rod shape have better bonding ability [19].

Dissipative particle dynamics (DPD) simulations can precisely model hydrodynamic interactions at a mesoscopic scale with acceptable time scales [22, 23], which can overcome the limitations of molecular dynamics simulations [24, 25] to predict complex hydrodynamics with much higher efficiency. Although DPD was first introduced to simulate the dynamics of fluids [26–28], it has been successfully used to reproduce hydrodynamic forces [27], explore the phase behavior of lipid molecules [29], and study the interactions of biomembranes and NPs [30–33]. For example, Filipovic et al. [34] used DPD to simulate the motions of circular and elliptical particles in 2D shear flow and compared their results with those obtained from finite element (FE) calculations to validate the ability of the DPD method to model the motion of micro/nanoparticles at the mesoscale. They also combined the multiscale mesoscopic FE bridging procedure with DPD and the lattice Boltzmann method to model the motion of circular and elliptical particles in 2D laminar flow [35]. This approach proved to be an effective way to model the motion of NPs in drug delivery systems. Meanwhile, Ding et al. [36] studied the effects of the coating ligands on the cellular uptake of NPs and found that the strength of the receptor–ligand interaction along with the density, length, and rigidity of the ligand can markedly affect the final equilibrium in receptor-mediated endocytosis.

Despite these exciting advances, theoretical modeling using approaches such as Brownian adhesive dynamics can provide some insights into adsorption kinetics and the dynamics of adsorbed NPs but lacks specific details about the binding process [37, 38]. This paper presents the details of dynamic transportation and adhesion of NPs to a vascular wall under shear flow determined using DPD simulations. Parameters such as bonding time and the mean-square displacement of NPs are considered. Results obtained for spherical NPs with different binding forces and diameters and for NPs with different shapes or aspect ratios but the same volume are compared to assess the influence of such parameters on the binding of NPs to a vascular wall.

## Methods

### Coarse-Grained (CG) Model: DPD Simulation

**Fig. 1:**
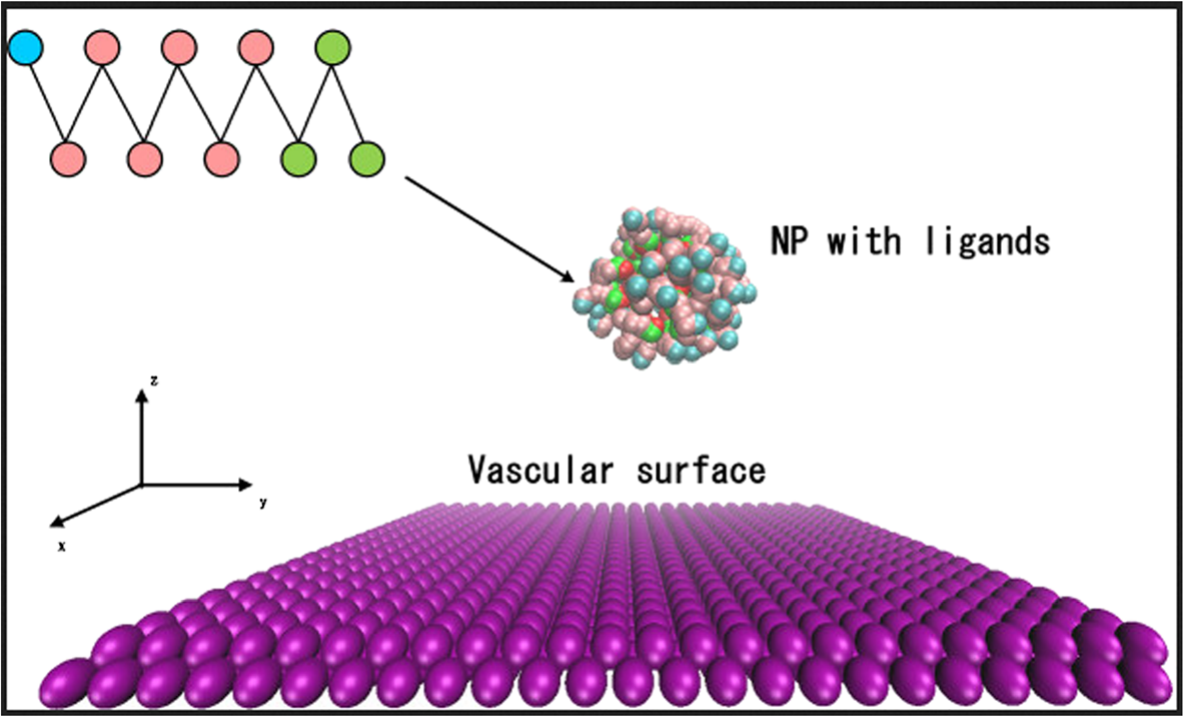
Coarse-grained model of a spherical nanoparticle (NP) and capillary surface. The NP is coated with ligands, and the vascular surface is covered with receptors (receptor effect was included in the potential but not explicitly modeled). The *cyan beads* represent the functional ends of each ligand; the *pink beads* represent the head of each ligand (hydrophobic beads); and the *green beads* represent the tail of each ligand (hydrophilic beads) that are permanently attached to the NP. The structure of the chains is magnified in the inset. Solvent molecules are omitted for clarity

To achieve targeted drug delivery, NPs are usually coated with polymers that specifically bind to a particular type of receptor on the vessel cell surface [37, 38]. It is computationally expensive to model the transportation and adhesion processes using an atomistic molecular dynamics simulation. However, the coarse-grained (CG) method guarantees that the general trend of the simulation will be determined without entirely erasing the structural details [39]. In this work, CG models were used to represent the components within the simulation system. The ligands on each NP were modeled as a chain of ten coarse-grain beads, namely, three hydrophobic beads and seven hydrophilic beads connected linearly to represent the polar head groups. As shown in Fig. 1, chains were uniformly distributed on the surface of a spherical NP. A harmonic potential was used to model the diblock copolymer chain, and the spring constant was set to 100 (reduced DPD units).

The size of the simulation box in our work was 22*r*_c_ × 22*r*_c_ × 22*r*_c_ (*r*_c_ is interaction length) with periodic boundary conditions in the *x* and *y* directions. The vascular surface was simplified as a fixed wall and placed at the boundary of the system consisting of fixed CG beads during the simulation. The wall was impenetrable with a “no slip” boundary condition where both the normal and tangential components of the particle momentum were inverted [40]. During the whole process, the wall particles did not move, acting as the location of the receptor that could interact with the ligands on the NPs. NPs with a diameter of 2 nm were also modeled by rigid beads placed in the middle of the box and filling the rest of the space with 27,783 explicit fluid particles [41]. The number densities *ρ* of the vascular wall and fluids were set as 3, as suggested elsewhere [42].

### Interaction Forces and Units in DPD Simulations

The interaction forces between different beads in the DPD formulation include a conservative force *f*^*C*^, a bead-spring force of the bonded monomers *f*^S^, a dissipative force *f*^D^, and a random force *f*^R^ [43]:

1 $$ \begin{array}{l}{f}_{ij}={f}_{ij}^{\mathrm{C}}+{f}_{ij}^{\mathrm{S}}+{f}_{ij}^{\mathrm{D}}+{f}_{ij}^{\mathrm{R}}\hfill \\ {}\kern1.12em =\left[-{a}_{ij}\left({r}_{\mathrm{c}}-{r}_{ij}\right)-K\left({r}_{\mathrm{s}}-{r}_{ij}\right)-y{w}^{\mathrm{D}}\left(\overrightarrow{r}/{r}_{ij},{v}_{ij}\right)+\sigma {w}^{\mathrm{R}}{\left({\varsigma}_{ij}\left(\varDelta t\right)\right)}^{-0.5}\right]\overrightarrow{r}/{r}_{ij},{r}_{ij}<{r}_{\mathrm{c},}\hfill \end{array} $$

where *a*_*ij*_ is the repulsion factor, *r*_*ij*_ and *v*_*ij*_ are the respective distance and velocity vectors of particle *i* with respect to particle *j*, *r*_c,_ and *r*_s_ are the respective cutoff distances for conservative and bead-spring forces, *K*, *γ*, and *σ* denote the spring constant, friction coefficient, and noise amplitude, respectively, *ω*^D^ and *ω*^R^ are the weight functions (*ω*^D^ = (*ω*^R^)^2^ = (*r*_c_ − *r*_*ij*_)^2^), *ɛ*_*ij*_ is the Gaussian random number, and Δ*t* is the simulation time step.

The random noise strength is expressed as a function of the dissipation strength and temperature *T* via the fluctuation–dissipation relation [28],

2 $$ {\sigma_{ij}}^2=2{\gamma}_{ij}{k}_{\mathrm{b}}T $$

where *σ*_*ij*_ and *γ*_*ij*_ are the random noise strength and dissipation strength between beads *i* and *j*, respectively. We carried out the simulations using a frictional coefficient *γ* of 3.

The default interactions between elements in DPD were described by the repulsion factor *a*_*ii*_ = 25*k*_b_*T*/*r*_c_ to guarantee the compressibility of water at room temperature. Interaction factors between hydrophobic and hydrophilic beads were set to 45, while the others were set to 25 [44]. The repulsion factors between different elements used in the DPD simulation are listed in Table 1, where FE, HL, TL, VS, and WM denote the functional end of the ligand, the head of the ligand, the tail of the ligand, the vascular surface, and a water molecule, respectively.

**Table 1:** Repulsion factors between elements used in DPD simulations

	Repulsion factor					
Element	FE	HL	TL	NP	VS	WM
FE	25	45	25	25	5	25
HL		25	45	45	45	45
TL			25	25	25	25
NP				25	25	25
VS					25	25
WM						25

To implement DPD simulation, *r*_c_, the bead mass *m*, and the thermostat temperature *k*_b_*T* were set as unit elements [43, 45, 46]. All simulations were performed in the *NVE* ensemble with constant particle number *N*, simulation box volume *V*, and energy *E*. The velocity Verlet algorithm was used to integrate with a relatively small time step of Δ*t* = 0.02*τ*, and each simulation was run for 4 × 10^5^ steps.

### Simplified Shear Rate

To apply shear flow in the flow region, we employed the SLLOD algorithm [47, 48] using the following equations:

3 $$ \frac{d{r}_{i,v}}{dt}=\frac{P_{i,v}}{m_v}+y{z}_v{\delta}_i, $$

4 $$ \frac{d{p}_{i,v}}{dt}={F}_{i,v}-\overset{\bullet }{y}{p_{z,}}_v{\delta}_i, $$

where *r*_*i*,*v*_, *p*_*i*,*v*_, and *m*_*v*_ are the position vector, peculiar momentum, and mass of the *v*th bead, respectively, *y*^•^ is the shear rate, and *δ*_*i*_ is the unit vector in the *x* direction. This approach allows us to impose a linear velocity profile in the *x* direction with a constant gradient in the *z* direction.

### Statistical Analysis

**Fig. 2:**
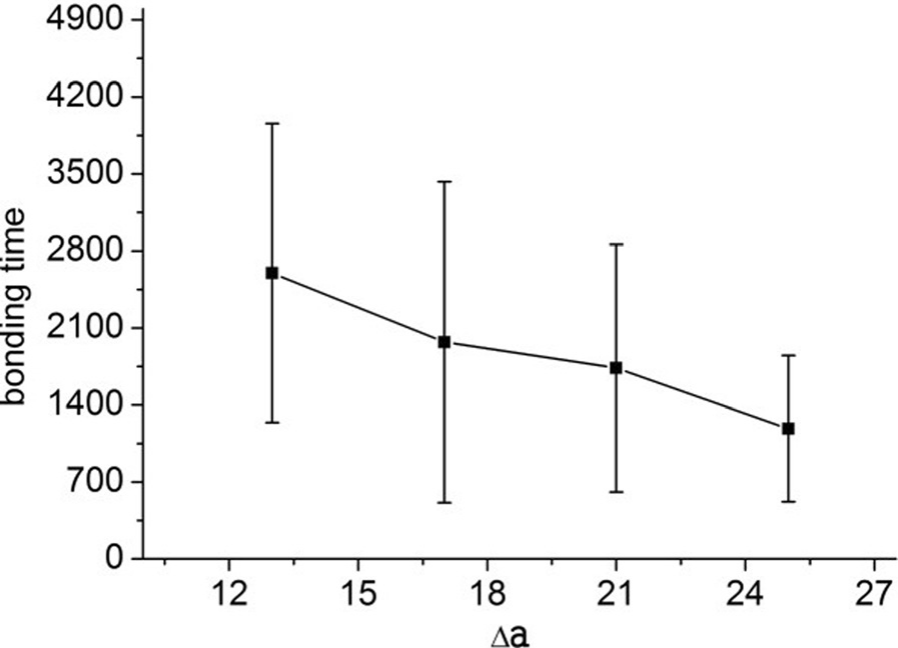
Bonding time for the attachment of NPs with strong binding strength. Bonding time required from the beginning of the simulation to firmly bond 2-nm NPs to the wall under a shear flow of 1000 s^−1^ when 13 < Δ*a* < 25

**Fig. 3:**
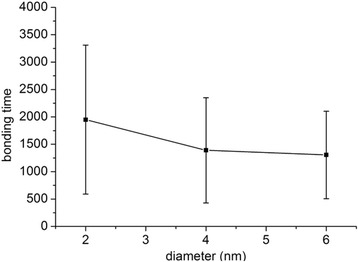
Mean and standard deviations of bonding time for NPs of different sizes. Data for NP diameters of 2, 4, and 6 nm are presented

**Fig. 4:**
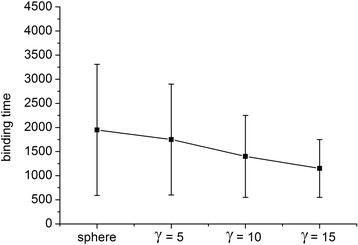
Mean and standard deviations of bonding time for NPs of different shapes. Shapes include spherical NPs and nanorods with *γ* of 5, 10, and 15, Δ*a* = 20

We examined the significance of the data presented in Figs. 2, 3, and 4 below. All *P* values were <0.05, so these data are significant at 0.05 level, indicating that it is highly unlikely that these results would be observed under the null hypothesis, and bonding times for different *x* values are significantly different. Therefore, even though the error bars in these figures look wide, the results are reasonable.

## Results and Discussion

**Fig. 5:**
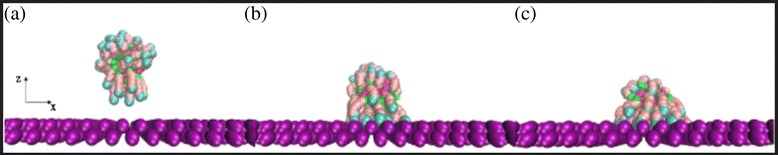
Bonding process of a spherical NP. NP (2 nm) under shear flow of 1000 s^−1^ when Δ*a* = 20. **a** *t* = 1800. **b** *t* = 1850. **c** *t* = 1900. Solvent molecules are omitted for clarity

The bonding processes of NPs under different shear flows were simulated with the developed CG model. A typical NP bonding process is shown in Fig. 5. As illustrated in Fig. 5a, the functional ends of an NP sense the attraction force from the vascular surface, and the ligands start to move toward the wall surface. Then, the NP is attracted to the wall until it is firmly attached to it, as shown in Fig. 5b, c.

### Effect of Binding Energy on Ligand–Receptor Binding Kinetics

Experimental results have shown that binding energies from 5 to 35 *k*_b_*T* strongly influence ligand–receptor binding kinetics [49]. In DPD simulation, the binding factor Δ*a* is defined as the difference between the ligand–receptor repulsion factor and the receptor–solvent repulsion factor. To simulate different attractive forces between ligands and the vascular surface, we varied the ligand–receptor repulsion factor while keeping the receptor–solvent repulsion factor constant. Here, Δ*a* = 5 indicates weak binding and the ligand–receptor repulsion factor is very close to that of the ligand with solvent, while Δ*a* = 25 indicates strong binding and the ligand–receptor repulsion factor is close to zero [44].

**Fig. 6:**
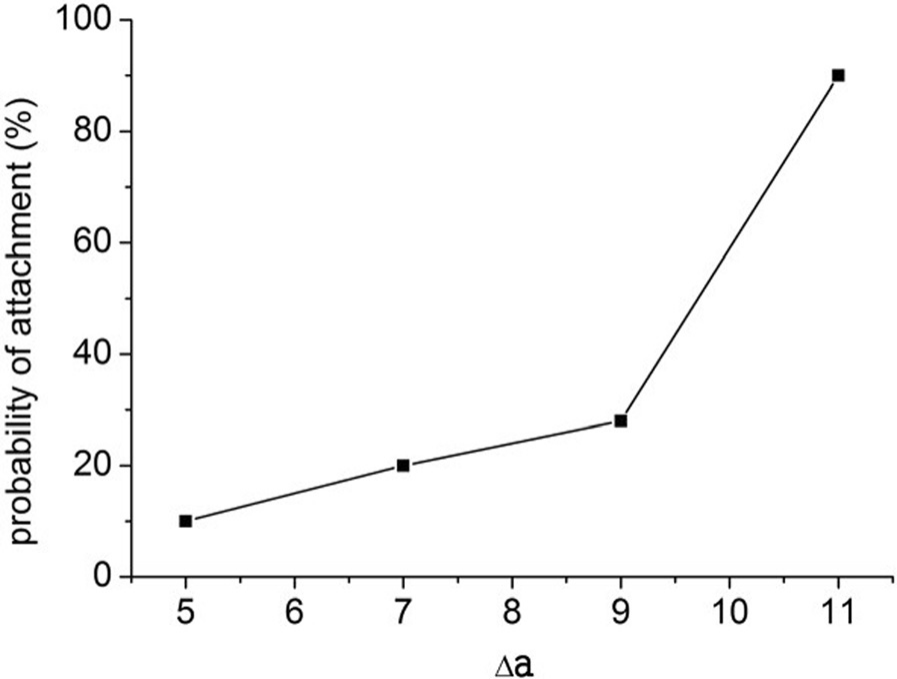
Probability of attachment of NPs with weak binding strength. NP (2 nm) under shear flow of 1000 s^−1^ when 5 < Δ*a* < 11

For relatively weak binding strength (5 < Δ*a* < 11), NPs mostly lingered in the middle of the flow domain and only a fraction of them established a stable contact with the receptor surface. For relatively strong binding strength (13 < Δ*a* < 25), bonding readily occurred and the binding time (from the beginning of the simulation to stable attachment) was measured. Figure 6 reveals that the probability of attachment initially increased linearly from about 10 to 30 % when 5 < Δ*a* < 9, and then increased abruptly to nearly 100 % when Δ*a* = 11. Figure 2 depicts ten different simulations run using various binding strengths. The mean bonding time decreased almost linearly as Δ*a* increased. The standard deviation of bonding time for each Δ*a* also decreased as Δ*a* increased. Therefore, NPs with a large bonding force have a higher probability of bonding and take less time to bond than those with a small bonding force. These results agree well with a previous report [44], which stated that when Δ*a* ≈ 12 or larger, any initial contact between ligands and a vascular surface leads to stable attachment.

### Effect of Shear Flow on the Binding Process

**Fig. 7:**
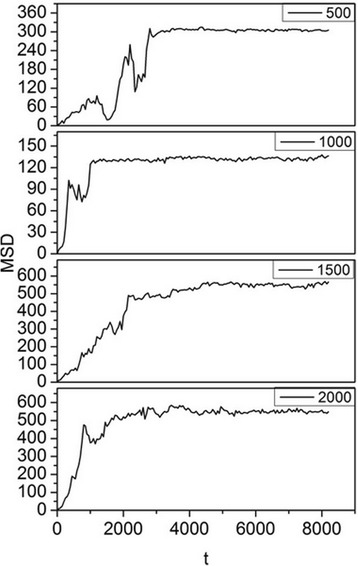
MSD as a function of bonding time. The *y*-axis is the MSD of the NP, and the *x*-axis is the corresponding time. Shear rates of 500, 1000, 1500, and 2000 s^−1^ with Δ*a* = 13 for 2-nm NPs are considered

The physiological range of shear rate in blood flow is approximately 40–2000 s^−1^, including flow within postcapillary venules, large arteries, and arterioles/capillaries [50]. In this paper, simulations were carried out for shear rates ranging from 0 to 2000 s^−1^ to study the NP bonding process under different shear flow conditions. The mean-square displacement (MSD) of 2-nm NPs under different shear rates was determined, and the results are shown in Fig. 7.

To simplify the analysis, we set Δ*a* = 13, which meant that the NPs would readily attach to the wall and the effects of the adhesion force can be minimized. In Fig. 7, the trajectory of NPs can be traced from their MSD. Initially, an NP moves randomly in the middle of the box under the influence of shear force and Brownian motion, and thus, the curve rises and falls at the outset. When the NP moves near the wall, it is attracted by the receptors, so it progresses to the wall and is bound to it, at which point the curve reaches the maximum MSD. After binding, the NP can still move because of the drive velocity [44] originating from the Brownian movement. The chains of each NP are long enough to allow reasonable vibration of the NP. Therefore, the curve subsequently fluctuates around the ultimate MSD.

**Fig. 8:**
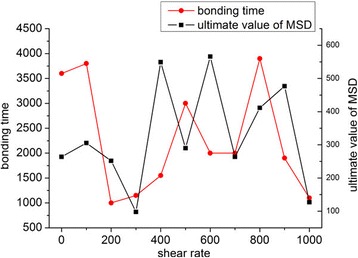
Effect of shear flow on bonding time and MSD. For 2-nm NPs with Δ*a* = 13

Higher shear rate is reported to result in lower bonding possibility for NPs when the NP diameter is larger than 100 nm [51]. However, we found that the bonding efficiency (time needed for the NPs to reach equilibrium) did not decrease with increasing shear rate, as seen in Fig. 7. The bonding situation may be different for NPs with a diameter of 2 nm in fluid at a position 20 nm above the capillary wall, so we compared the bonding time and ultimate MSD of NPs for shear rates ranging from 0 to 2000 s^−1^, as shown in Fig. 8. With increasing shear rate, the bonding time and ultimate MSD do not decrease accordingly. Instead, the spots appear randomly in relation to shear rate, which means that the shear rate has no effect on the bonding process in a certain area. Even when the shear rate was set to 0 s^−1^, the bonding time was still longer than that in most cases with shear rate. This is because Brownian force outweighs the drag force and is the dominant force for NPs larger than 100 nm [51], a phenomenon that is even more obvious for smaller NPs. This is the reason for the heterogeneous binding time and MSD in Fig. 8. As a result, for NPs with a diameter of 2 nm, the bonding probability is not influenced by shear rate. NPs will firmly bond to the wall once in contact with it, and thus, bonding condition is dominated by diffusive process and independent of shear flow. We also simulated the behavior of NPs with diameters of 4 and 6 nm under the same conditions and obtained equivalent results.

### Effect of NP Size on the Binding Process

**Fig. 9:**
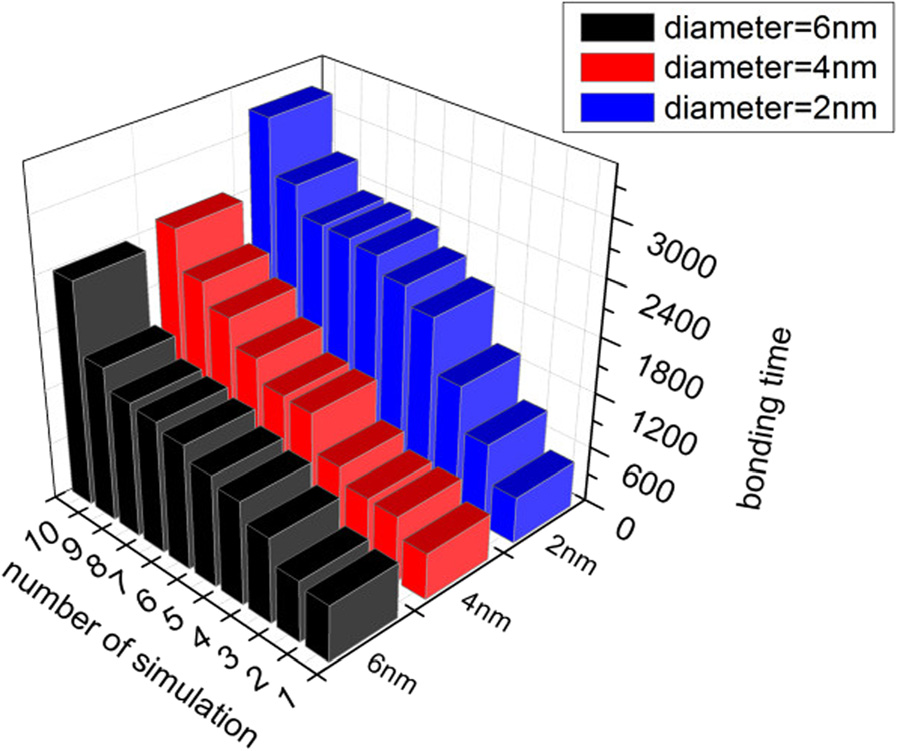
Effects of NP size on the binding process. Bonding times of NPs with diameters of 2, 4, and 6 nm when Δ*a* = 20; data are sorted in ascending order

The Brownian force $\left({F}_{\mathrm{B}}\propto {R}^{\frac{1}{2}}\;\mathrm{f}\mathrm{o}\mathrm{r}\;\mathrm{r}\mathrm{o}\mathrm{und}\;\mathrm{nanoparticle}\;\mathrm{in}\;\mathrm{f}\mathrm{luid}\right)$ will increase with the size of the NPs. Therefore, NPs with diameters of 4 and 6 nm were also simulated to study how NP size affects its bonding process. The size limitation of the simulation box meant that 6 nm was the largest size of NP we could investigate here. The bonding times of NPs with diameters of 2, 4, and 6 nm are illustrated in Fig. 9. The results indicate that the bonding time is shorter for larger NPs.

To allow quantitative analysis, the mean and standard deviations of bonding time for NPs of different sizes are presented in Fig. 3. Figure 3 reveals that the average bonding times are shorter and the standard deviations smaller for larger NPs. In our simulation, Brownian force is the determinative force, indicating that the motion of small particles in fluids is controlled by random collisions with surrounding fluid molecules. The random collision of NPs of smaller size is less likely to be balanced than that of larger NPs. If the unbalanced force is not in the direction of the wall, which is more likely to happen than the force being in the direction of the wall, the NP is less likely to be attracted to the wall and its bonding time will be prolonged. The track of a smaller NP is more disordered than that of a larger NP because of the unbalanced random force, which results in a larger standard deviation. Thus, the simulation results demonstrate that a bigger NP has higher bonding ability than a smaller one.

### Effect of NP Shape on the Binding Process

**Fig. 10:**
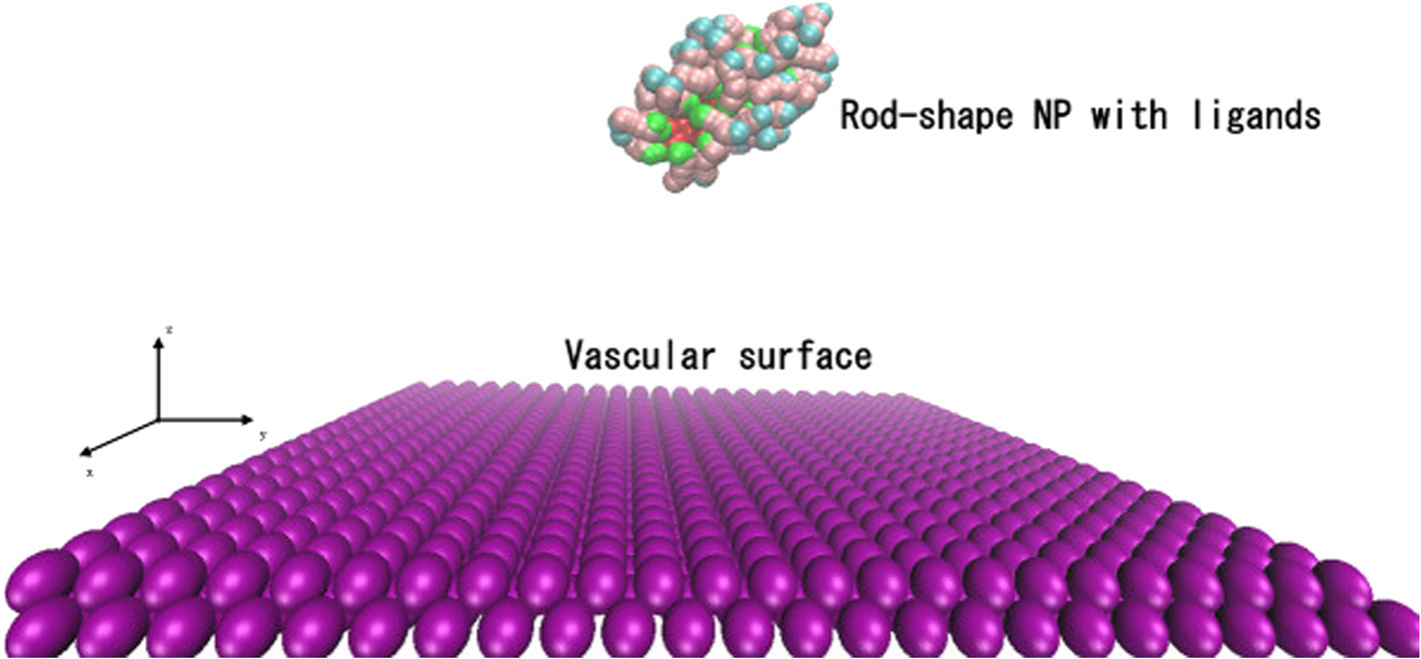
Coarse-grained model of a nanorod and capillary surface. The nanorod has the same volume and ligands as the 2-nm spherical NP, where *γ* = 3. Solvent molecules are not shown for clarity

Both simulation and experimental results show that shape strongly affects pharmacokinetics and pharmacodynamics [52]. Different shapes cause contact areas, random forces of Brownian motion, and drag forces induced by shear flow to vary. Therefore, rod-shaped NPs (nanorods) were investigated in addition to spherical NPs to study the effect of NP geometry on the particle bonding process [38]. Nanorods with aspect ratios (*γ*; ratio of long axis to short axis) [51] of 5, 10, and 15 were considered. Nanorods with *γ* = 3 is shown in Fig. 10. The volume of the nanorods used was the same as that of the 2-nm NPs to ensure the same drug load capacity.

**Fig. 11:**
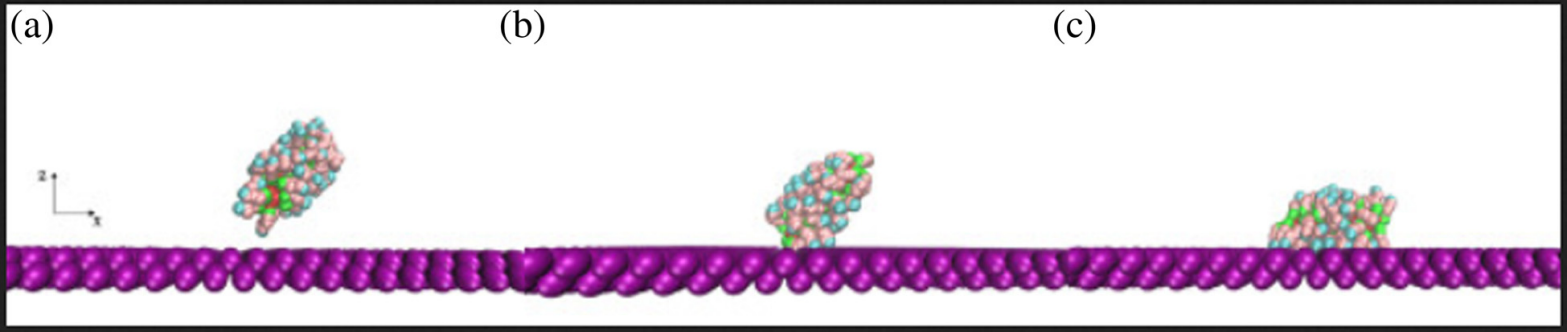
Bonding process of a nanorod. Δ*a* = 20 and *γ* = 3. **a** *t* = 1500. **b** *t* = 1550. **c** *t* = 1600. Solvent molecules are omitted for clarity

Figure 11 illustrates the bonding process of a nanorod. In Fig. 11a, the ligands on the end of the nanorod start to sense the attraction force from the vascular surface. Then, the ligands on the side of the nanorod are gradually absorbed by the wall until they are firmly attached to it, as depicted in Fig. 11b, c.

**Fig. 12:**
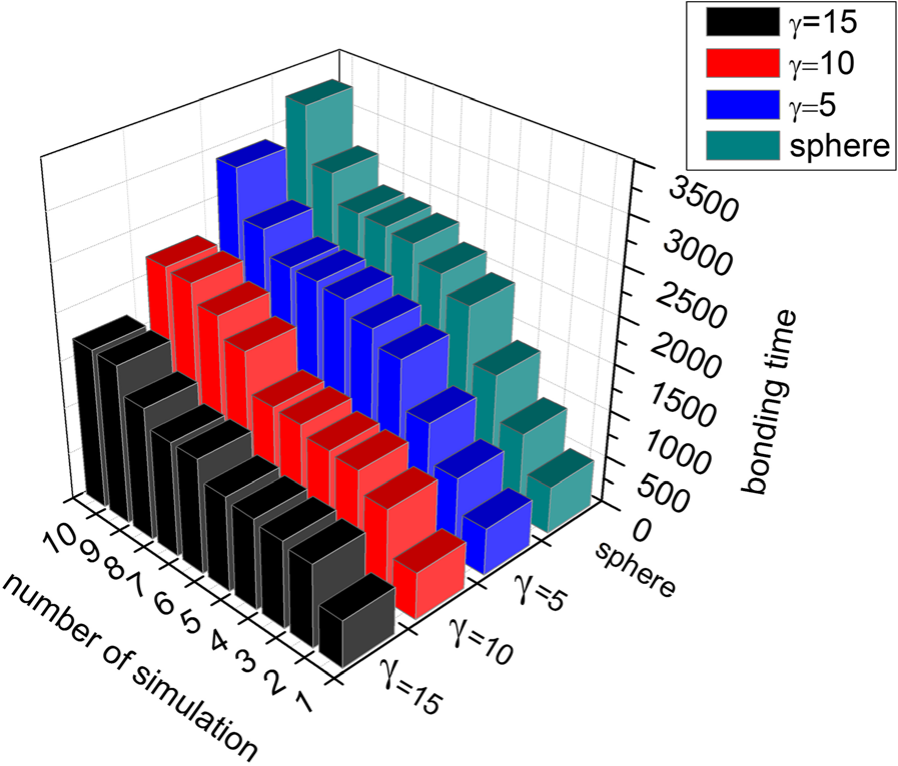
Effects of NP shape on the binding process. The bonding times of a spherical NP and nanorods with *γ* of 5, 10, and 15 were measured. The NP volume was kept constant; data are sorted in ascending order

Figure 12 compares bonding times for NPs of different shapes. Bonding efficiency was higher for nanorods than spherical NPs, and increased with *γ*. Figure 4 reveals that the average bonding time and standard deviation were smaller for nanorods than spherical NPs. This is because of the tumbling motion and the larger contact area of the nanorods compared with the spherical NPs [38]. The contact area of a spherical NP is irrelevant to its orientation, and the binding area remains constant within interacting distance. Conversely, for nanorods, the contact area depends on orientation, and there is a higher chance to initiate contact with the wall because of its longer length compared with spherical NPs. Both the mean and standard deviations of bonding time decrease with increasing *γ* because NPs with a larger *γ* are thinner and longer, so the ligands on the end of the nanorod have a higher chance of interacting with the wall. These results are consistent with the finding that the strength of adhesion increases with *γ* [53], and thus, the bonding time decreases.

## Conclusions

We used DPD simulations to study the dynamics of polymerized NP binding with a vascular surface. We described in detail how the shear rate, bonding energy, size, and shape of an NP affect its bonding ability. The results indicate that the bonding ability increases linearly with bonding energy. Interestingly, the shear rate does not influence the bonding process of NPs with a diameter of 2–6 nm in a liquid environment 20 nm above the capillary wall. Compared with small spherical NPs, those with larger diameter or rod shape will move in a more orderly manner and require less time to reach the surface of the capillary wall, which means that they have better bonding efficiency. Additionally, the bonding ability of nanorods increased with *γ*. Our results provide some useful theoretical bases for designing NPs, which may aid in the development of new types of NPs with advantageous functionalities for biomedicine applications.

## Abbreviations

CG: Coarse-grained
DPD: Diffusive particle dynamics
FE: Finite element
NP: Nanoparticle
